# Analysis of β-nerve growth factor and its precursor during human pregnancy by immunoaffinity-liquid chromatography tandem mass spectrometry

**DOI:** 10.1038/s41598-023-34695-7

**Published:** 2023-06-06

**Authors:** Jason Walsh, Joe Palandra, Eduward Goihberg, Shibing Deng, Susan Hurst, Hendrik Neubert

**Affiliations:** 1 Pfizer Inc., 1 Burtt Road, Andover, MA 01810 USA; 2 Pfizer Inc., 1 Portland St, Cambridge, MA 02139 USA; 3 Pfizer Inc., 10777 Science Center Drive, San Diego, CA 92121 USA; 4 Pfizer Inc., 445 Eastern Point Road, Groton, CT 06340 USA

**Keywords:** Biochemistry, Chemical biology, Developmental biology, Neuroscience, Physiology, Biomarkers, Neurology, Chemistry

## Abstract

β-Nerve growth factor (NGF) is a neurotrophin that plays a critical role in fetal development during gestation. ProNGF is the precursor form of NGF with a distinct biological profile. In order to investigate the role of NGF and proNGF in pregnant human females, a sensitive and selective immunoaffinity liquid chromatography-tandem mass spectrometry assay was developed and qualified to simultaneously measure the levels of total NGF (tNGF; sum of mature and proNGF) and proNGF using full and relative quantification strategies, respectively. The assay was used to determine serum tNGF and proNGF levels in the three gestational trimesters of pregnancy and in non-pregnant female controls. Mean tNGF ± SD were 44.6 ± 12.3, 42.6 ± 9.3, 65.4 ± 17.6 and 77.0 ± 17.8 pg/mL for non-pregnant, first, second, and third trimesters, respectively, demonstrating no significant increase in circulating tNGF between the control and the first trimester, and a moderate yet significant 1.7-fold increase through gestation. proNGF levels during the first trimester were unchanged compared to control. In contrast to tNGF, however, proNGF levels during gestation remained stable without significant changes. The development of this sensitive, novel immunoaffinity duplexed assay for both tNGF and proNGF is expected to enable further elucidation of the roles these neurotrophins play in human pregnancy as well as other models.

## Introduction

β-Nerve growth factor (NGF) and its precursor proNGF are important neurotrophins that have differing functions. NGF promotes neuronal growth, development and differentiation and is generally neuroprotective^1^, while proNGF has seemingly contradictory functionality^2^, and has been found overexpressed in thyroid cancers compared to normal^3^. Reports published indicate both support for the growth and survival of neurons^4,5^, as well as neuronal death^6,7^. proNGF is the dominant form that exists in the brain, with little to no mature NGF present^8,9^. Interestingly the role of the ratio of proNGF to mature NGF in neural cells^10,11^ and subsequently the role of receptor expression and signaling^8,12,13^ by these distinct proteins appears to be important factors in neurodegenerative conditions^14,15^. The two proteins exist in equilibrium, and the alteration of that balance may cause them to function antagonistically towards one another^16^. For example, defective maturation of NGF, thus resulting in a higher proNGF to mature NGF ratio in the brains of rats contribute to early progression of experimentally induced diabetic encephalopathy^17^. Furthermore, an imbalanced ratio between proNGF and mature NGF seems to be an early indicator for complications from diabetes generally^18^. Improper regulation or disfunction of NGF may also be implicated in neurodegenerative disorders such as Alzheimer’s disease^19–22^, age related macular degeneration^23–26^, retinal injury^27^, and multiple sclerosis^28^, and NGF has also been investigated for a therapeutic role in the neurodegenerative disease glaucoma^24^. Since its discovery and isolation^29,30^, it is recognized that NGF has effects on the development of nervous systems^31–33^ and plays a key role in adult nociceptive modulation^34,35^. Therefore, NGF has been widely studied for its therapeutic significance and is increasingly pursued as a potential therapeutic target in nociceptive pain and oncology^36,37^.

Neurotrophins are also involved in processes related to pregnancy. Both maternal and fetal NGF expression has been shown to be elevated in the first through third trimesters of pregnancy implicating an important role in both egg implantation and increasing maternal vascularity in the late stages of pregnancy^38,39^. NGF supplementation has been shown to increase endometrial vascularization in certain mammals^40^, which could explain a vascularity compensation for elevated NGF in placental circulation in preeclampsia births^41^, while lower cord plasma and placental NGF levels are associated with preterm births^42^. Furthermore, lower NGF levels due to an outside factor such as opioid addiction is associated with significantly higher adverse pregnancy outcomes including cognitive anomalies, neonatal death, respiratory problems, and lower Apgar scores^43^.

The ratio of proNGF to mature NGF may also play a similar role to that in neurodegenerative disorders as outlined above, but likely is responsible for the breakdown of uterine sympathetic nerves during gestation, and the re-innervation of uterine nerves during postpartum recovery^44,45^, and unbalanced NGF distribution in placental tissue is associated with miscarriages in humans^46^. NGF levels were shown to be elevated in human neonates at day 4 compared to umbilical cord blood (after an initial decrease at day 1), while other neurotrophins decreased significantly^47^. Along with the positive associations of specific interleukins with insulin resistance and secretion, NGF was higher in the gestational diabetes patients and strongly linked to glucose metabolism, insulin resistance and pancreatic β cell function in Chinese pregnant women in the second trimester.

Previous work using the LCMS assay for NGF demonstrated a large and continuous increase in NGF expression (up to ~ 78×) during gestation in cynomolgus monkeys, with no difference in circulating levels between male and the non-pregnant female control populations^48^. Though a large increase in circulating tNGF levels has been shown in cynomolgus monkeys^48^, this phenomenon has not been reported in human pregnancy which seems to point towards species specific differences during pregnancy. While a balance of systemic NGF levels are important for optimal pregnancy progression, the NGF mRNA expression in human normal pregnancy versus spontaneous abortive patients from isolated trophoblasts showed a ~ 2 to 8 fold increase in the latter. This result was consistent with spontaneous abortion in mouse models when a supraphysiological dose of NGF was administered in early pregnancy^46^, and NGF levels have been shown to increase in post-gestational lactating mice^49^. Intriguingly, NGF levels in rat uterus are reduced during middle and late pregnancy compared to controls, which does correlate with known myometrial nerve degeneration^45^. Similarly, NGF expression is elevated in diabetes mellitus and it was shown to be a protective factor in diabetic neuropathy and vasculopathy^50–53^, but was also elevated and implicated in insulin resistance in gestational diabetic Chinese women in the second trimester^54^. Lobos et al., also demonstrated that proNGF levels increase during gestation possibly due to impaired processing of the pro form to the mature form^44,45^.

Collectively, the aforementioned studies have advanced the knowledge of the various roles of NGF and proNGF during pregnancy. However, the NGF and proNGF analysis by and large were conducted without qualified, selective assays and the reported data sets seem both comparable, as well as divergent. Therefore, it remains unconfirmed if the reported differences in NGF and proNGF concentrations, and thus the conclusions reached, at least in some cases, may be confounded by assay differences and variability^16^. This emphasizes the need to characterize, potentially improve and qualify the proNGF and NGF assays to gain confidence in the conclusions^16^.

Utilizing a previously qualified immunoaffinity-liquid chromatography tandem mass spectrometry (IA-LC–MS/MS) approach for quantification of tNGF in human serum^55–57^, we extended the assay to include a relative quantification of proNGF. With this analysis using a highly selective and sensitive mass spectrometry assay, we intend to confirm tNGF levels during human gestation, and incorporate a relative quantitative measure of the precursor protein. The ability of the assay to discern proNGF enables the assessment of changes in proNGF serum levels during pregnancy. We used this approach to evaluate the tNGF and proNGF levels in the serum of pregnant women near the midpoint of each trimester compared to a non-pregnant female cohort. This data can now be used to further understand the role of NGF and proNGF during gestation in human pregnancy.

## Results

### tNGF/proNGF assay development

A surrogate peptide for proNGF was necessary to give an equimolar representation of the proNGF protein; that is for every 1 mol of proNGF, 1 mol of the surrogate peptide was required to be generated after treatment with trypsin. A tryptic peptide VLFSTQPPR derived from propeptide region of proNGF was selected based on specific criteria to meet the objectives of the assay as described herein: mainly the specificity to the proNGF peptide in a domain not present in the mature form of NGF; secondly, high predicted antigenicity allowing the generation of a specific antipeptide antibody; finally, the ability to achieve a robust signal in the mass spectrometer. The second tryptic peptide used in this study was IDTACVCVLSR, which was previously used in LC–MS/MS assays for NGF measurements The selection criteria for peptide IDTACVCVLSR have been previously described^55^. This peptide is from near the C terminus of both the mature NGF and proNGF proteins and therefore represents contributions from both protein forms in a combined tNGF measurement.

Necessary to the development of the assay, the capture antibody used for this assay would have to be able to bind both mature NGF and proNGF. The binding of the polyclonal capture antibody to both proteins is confirmed by specific detection of both surrogate peptides. Furthermore, prior to the qualification study, repeated probing of supernatants following antibody enrichment from human serum confirmed that the capture antibody has close to complete recovery of both protein forms.

### Assay qualification

**Figure 1:**
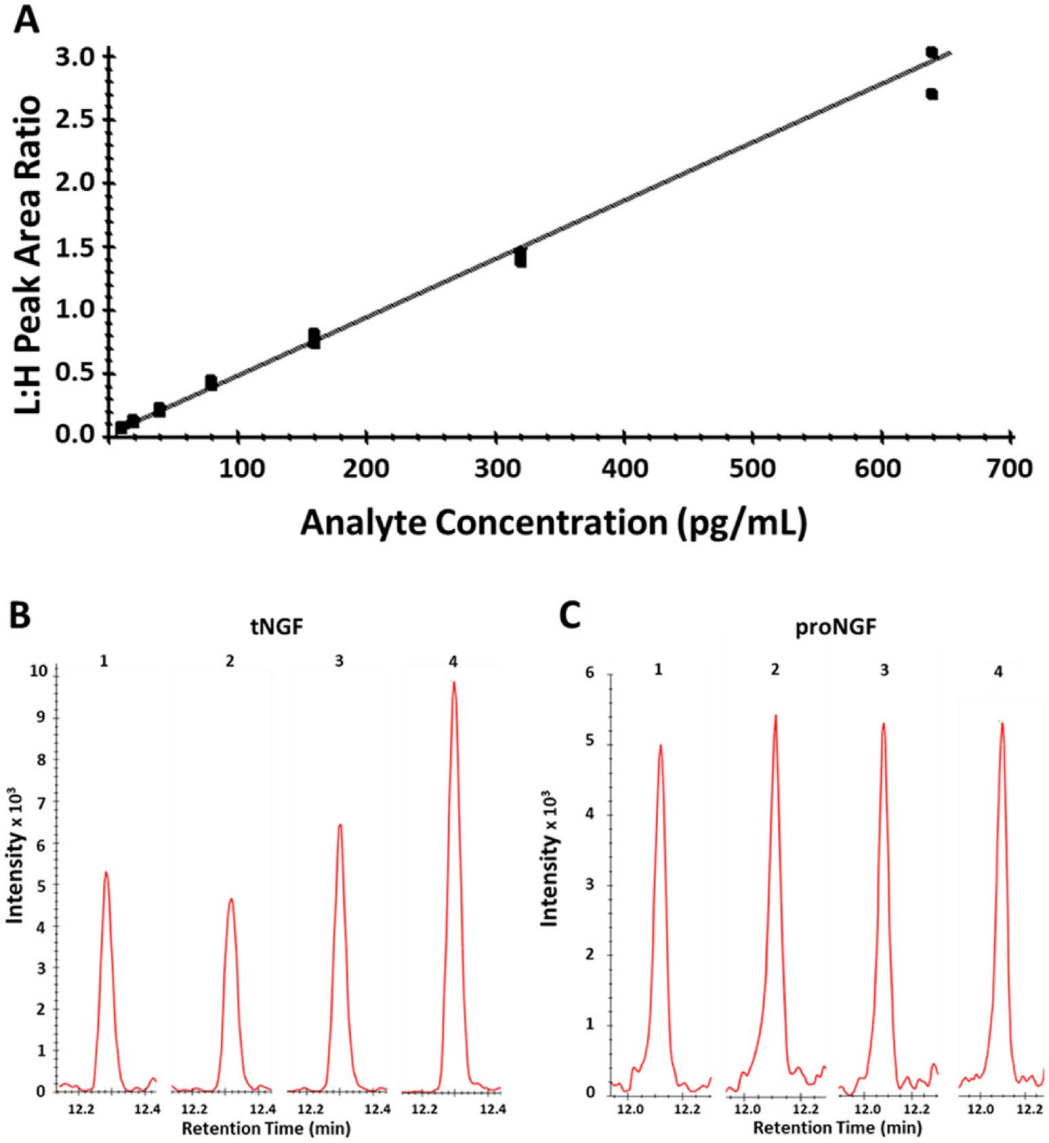
(**A**) Representative calibration curve for tNGF. (**B**) tNGF in pregnancy samples (1) control, (2) 1st trimester, (3) 2nd trimester, (4) 3rd trimester. (**C**) proNGF (1) control, (2) 1st trimester, (3) 2nd trimester, (4) 3rd trimester.

The method was qualified with three individual batch runs carried out on separate days. The range of quantification in human serum for tNGF is 10.0–640 pg/mL using relative accuracy acceptance criteria of < 25% (30% at LLOQ). Eight calibration standard concentration levels (including the 0-point) were represented in the final curves with a minimum of six non-zero points required. A typical calibration curve obtained during assay qualification is shown in Fig. 1A. The CV range for all calibration standards was 4.80 to 16.6%, and a RE range of − 5.20 to 12. 4%. Only four individual calibration points were removed from the calibration curves throughout the three separate batch runs. Out of acceptance individual curve points were removed and the curve fit re-regressed. tNGF precision and relative accuracy was assessed in the same serum lot throughout the qualification study which gave an inter-assay mean of 30.0 pg/mL (QC2; endogenous tNGF level), with the twofold dilution of QC2 (QC1) having an inter-assay mean of 12.8 pg/mL. For the spiked QCs, i.e., QC3 and QC4, the determined inter-assay mean for the endogenous level for NGF in QC2 was added to the spiked NGF concentration to calculate the relative accuracy. Individual, intra- and inter assay QC data and summary statistics for tNGF are shown in Table 1.

**Table 1:** Accuracy and precision for tNGF (pg/mL) during assay qualification.

tNGF												
Level	QC1			QC2			QC3			QC4		
Nominal	½ Endogenous			Endogenous			Endogenous + 45 pg/mL			Endogenous + 450 pg/mL		
Target tNGF level (pg/mL)	15.0			30.0			75.0			485		
Qual Run	1	2	3	1	2	3	1	2	3	1	2	3
Rep 1	9.92*	12.8	11.9	32.0	31.9	27.5	65.0	59.6	62.8	475	485	483
Rep 2	14.1	10.1*	14.6	32.0	30.3	27.6	74.2	60.8	67.4	434	380	524
Rep 3	15.8	11.0*	13.0	27.8	25.6	26.1	62.9	73.2	77.4	458	418	465
Rep 4	12.1	13.2	14.1	33.0	30.1	26.6	63.9	60.3	71.3	426	406	487
Rep 5	11.1*	14.9	10.5*	34.5	35.1	30.5	73.9	68.9	85.0	420	405	440
Rep 6	15.6	12.4	12.5	26.0	32.5	30.9	71.1	74.8	90.9	432	375	488
Avg	13.1	12.4	12.8	30.9	30.9	28.2	68.5	66.3	75.8	440.8	411.5	481.2
SD	2.44	1.69	1.49	3.27	3.17	2.02	5.16	6.90	10.72	21.17	39.60	27.84
CV %	18.6	13.6	11.7	10.6	10.3	7.16	7.53	10.4	14.1	4.80	9.62	5.79
RE %	−12.6	17.3	14.9	2.94	3.06	−6.00	−8.67	11.6	1.07	2.04	−8.56	6.93
Inter-Assay												
Mean	12.8			30.0			70.2			445		
SD	1.82			3.00			8.56			41.0		
CV %	14.3			10.0			12.2			9.23		
RE %	−150			NA			−6.41			−7.40		

All points included in statistical calculations.

*Qual* qualification, *Rep* replicate, *Avg* average, *SD* standard deviation, *RE* relative error.

*Accuracy > 25%.

For the spiked QCs, i.e., QC3 and QC4, the determined inter-assay mean for the endogenous level for tNGF in QC2 was added to the spiked NGF concentration to calculate the relative accuracy. Individual, and inter assay QC data and summary statistics for tNGF are shown in Table 1.

Precision and relative accuracy for proNGF was demonstrated over the same three batches used for tNGF qualification on three separate days at five different levels. The values shown in Table 2 are the peak-area-ratios (PAR) of light peptide to heavy peptide signal. The percent relative error (RE %), as a measure for relative accuracy, was calculated using the experimentally determined average PAR values for low and high QCs (proQCL and proQCH) and comparing experimental with extrapolated, theoretical PAR response for the admixing QCs (proQCM1, proQCM2 and proQCM3). Inter-assay mean, CV % and RE % for proNGF qualification QCs were proQCL 0.0699, 18.4% (No RE); proQCM1: 0.115, 25.6%, 19.1%; proQCM2: 0.131, 22.4%, 7.09%; proQCM3 0.156, 15%, 4.69%; proQCH: 0.175, 18.2% (No RE) respectively. The proNGF inter-assay CV range was 15.0–25.6%, with an RE range of 4.69–19.1%.

**Table 2:** Accuracy and precision for proNGF (L:H peak area ratio) during assay qualification.

proNGF															
Level	proQCL			proQCM1			proQCM2			proQCM3			proQCH		
% Composition	100% proQCL			75% proQCL 25% proQCH			50% proQCL 50% proQCH			25% proQCL 75% proQCH			100% proQCH		
Target proNGF level (L:H PAR)	NA			0.096			0.123			0.149			NA		
Qual run	1	2	3	1	2	3	1	2	3	1	2	3	1	2	3
Rep 1	0.081	0.055	0.081	0.119	0.075	0.14	0.173	0.093	0.172	0.178	0.123	0.152	0.179	0.141	0.201
Rep 2	0.076	0.053	0.086	0.114	0.072	0.135	0.134	0.097	0.151	0.155	0.124	0.153	0.146*	0.111	0.207
Rep 3	0.088	0.063	0.078	0.126	0.085	0.157*	0.138	0.103	0.126	0.178	0.132	0.172	0.215	0.158	0.196
Rep 4	0.061	0.062	0.071	0.142	0.076	0.118	0.167	0.09	0.147	0.168	0.131	0.148	0.194	0.142	0.202
Rep 5	0.082	0.045	0.067	0.134	0.084	0.166*	0.159	0.088	0.131	0.179	0.13	0.195	0.191	0.163	0.194
Rep 6	0.072	0.055	0.083	0.123	0.081	0.118	0.136	0.102	0.158	0.184	0.131	0.176	0.224	0.137	0.157
Avg	Avg	0.0767	0.0555	0.078	0.126	0.079	0.139	0.151	0.096	0.148	0.174	0.129	0.166	0.192	0.142
SD	SD	0.0094	0.0066	0.0073	0.0102	0.0053	0.0198	0.0172	0.0062	0.0171	0.0105	0.0039	0.0183	0.0277	0.0184
CV %	CV	12.3	11.8	9.4	8.1	6.7	14.2	11.4	6.5	11.6	6.1	3.1	11.0	14.5	12.9
RE %	–	–	–	17.40	2.20	20.0	9.00	3.30	9.10	2.40	6.70	1.20	–	–	–
Inter-assay															
Avg	0.0699			0.115			0.131			0.156			0.175		
SD	0.013			0.029			0.029			0.023			0.032		
CV %	18.4			25.6			22.4			15.0			18.2		
RE %	NA			19.1%			7.09			4.69			NA		

All points included in statistical calculations.

*Qual* qualification, *Rep* replicate, *Avg* average, *SD* standard deviation, *RE* relative error.

*Accuracy > 25%.

**Figure 2:**
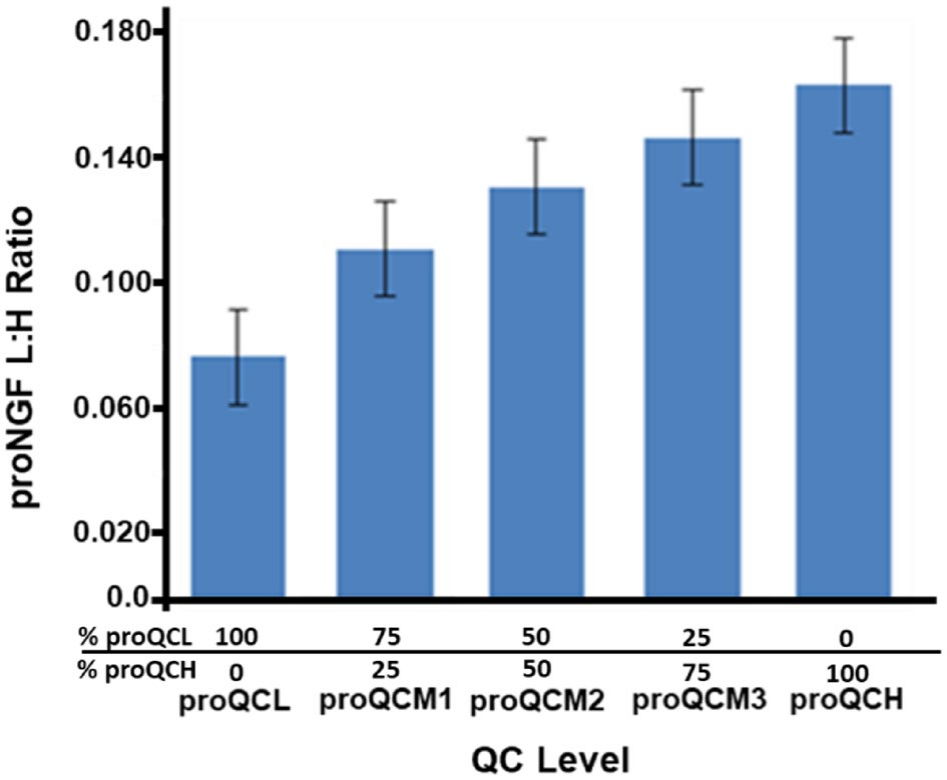
Mean proNGF L:H PAR (light: heavy peak area ratio) for all proNGF QCs generated by admixing proQCL with proQCH showing a 2.37-fold increase in proNGF L:H PAR from proQCL to proQCH.

We observed a 2.6, 2.5 and a 2.0 fold difference in the signals between the proQCH and the proQCL for qualification runs 1,2 and 3 respectively which gives a 2.37-fold average difference through the qualification (Fig. 2).

### Human pregnancy cohort results

During the analysis of the samples all tNGF values for QCs and unknowns were run in parallel on two 96-well plates with a four replicate calibration curve, and the QC samples divided between the two plates. Relative errors were calculated using the endogenous level measured for QC2 on the date of analysis, added to the amount of NGF spiked (Table S1). The proNGF QCs passed the acceptance criteria based on CV and relative error from the nominal concentration determined by the proNGF level measured for proQCL and proQCH on the date of analysis, using the mean of the proQCH and proQCL with the appropriate admixing scheme to calculate the theoretical ratio for proQCM2 (Table S2).

**Figure 3:**
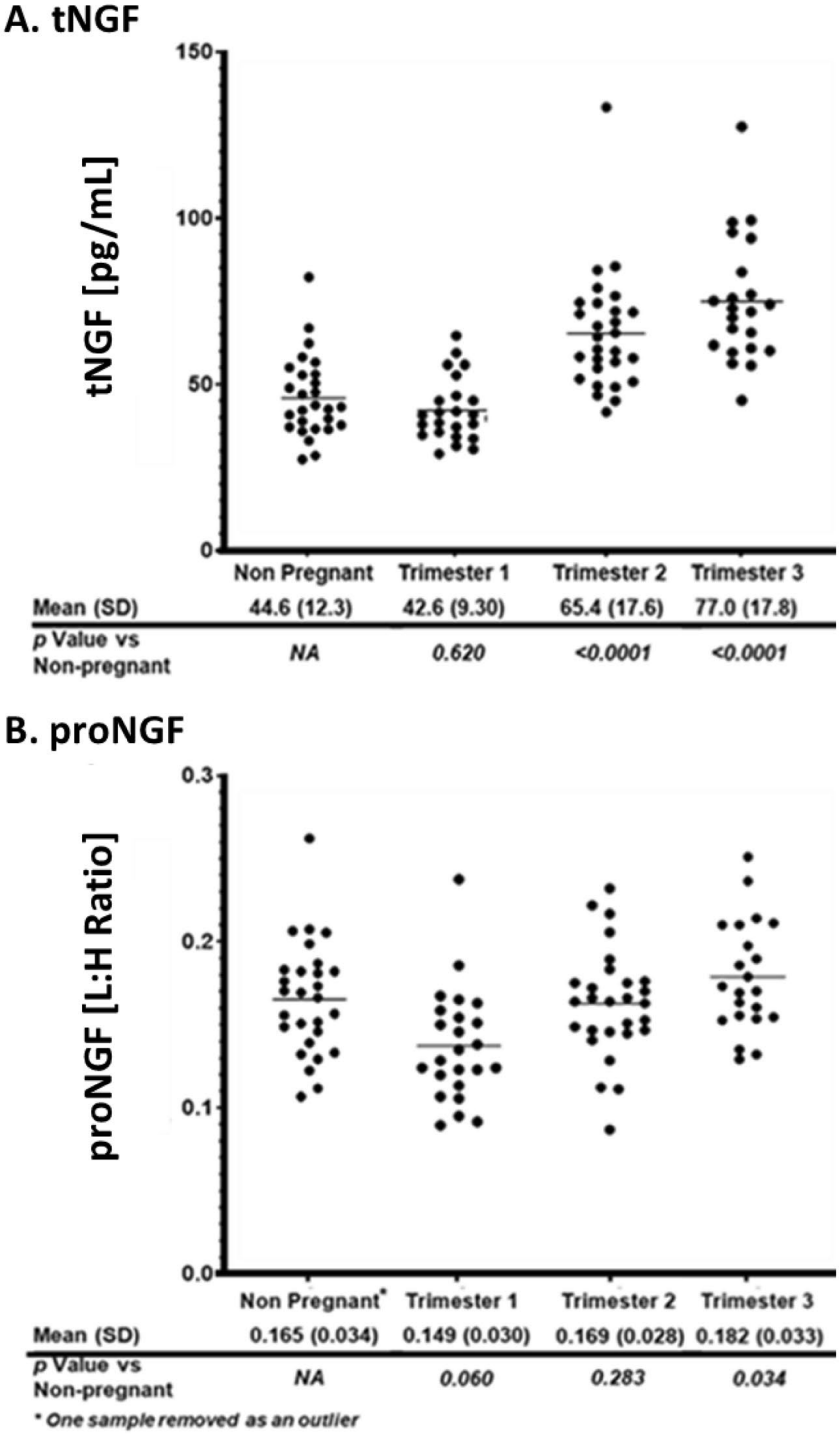
(**A**) tNGF and (**B**) proNGF levels in non-pregnancy and pregnancy cohorts broken down by trimester. Shown below each plot are the mean and standard deviation (SD) for each group as well as the *p* value as compared to the control (non-pregnant) group. Lines indicate the mean of each group.

Values for tNGF and proNGF in the cohort are grouped by trimester as well as the non-pregnant control (Fig. 3). The data shows significant increases in circulating tNGF levels from the control group to the second and third trimester, and no significant increase in circulating proNGF levels in those same groups. No covariates were found significantly associated with tNGF. We obtained age information from all 103 patients and an age-associated analysis was done on both peptides. Among 103 patients with tNGF data, the rank correlation between age of the individual subjects and tNGF is -0.153 with a *p* value of 0.126. The rank correlation between age of the individual subjects and proNGF is 0.124 with *p* value of 0.220. In a linear regression model, adjusting for trimester, there is no significant association between tNGF and age with a *p* value of 0.079; or between proNGF and age with a *p* value of 0.173 (Figure S1). Representative ion chromatograms for tNGF and proNGF from selected non-pregnant control, first, second and third trimester subjects are shown in Fig. 1B, C respectively. Individual values for the human pregnancy cohort for tNGF and proNGF can be found in the supplemental information (Table S3 and Table S4, respectively).

## Discussion

In this report we describe both the quantitative measurement of tNGF in human serum using IA-LC–MS/MS^55^ of a cohort of pregnant and non-pregnant women, as well as the novel relative quantification of proNGF utilizing the same methodology in a duplex assay format. Adapting the assay to a duplex format allowed for the measurement of both proteins in a single run, as the IA-LC–MS/MS assay requires only a single capture antibody reagent. The basal levels of serum tNGF measured in control serum was comparable to levels previously found via IA-LC–MS/MS^55^, and, although higher sensitivity ligand binding assay for NGF are in use, remained measurable to a tenfold greater sensitivity than some previously published ELISA studies^58^.

Here we demonstrate that mean tNGF levels do not change significantly between the non-pregnant control and the first trimester. However, the levels increased from the first trimester to the second, and again albeit more modestly from the second to the third (1.5-fold and 1.1-fold respectively) but with statistical significance based on *p* value (Fig. 3). The mean overall increase from the final trimester of pregnancy compared to the non-pregnant control group was a 1.7-fold and was significant, based on the p-values. This increase was greater than the observed variability established during assay qualification. Previous studies have shown small, non-significant changes in circulating mature NGF levels during human gestation^59,60^ and while the data contained in this study does show a small yet significant increase in tNGF over gestation, it is in general agreement with values published previously^48,59^.

Based on the *p* values, proNGF levels did not significantly change over the course of gestation. Circulating proNGF levels (or cleaved derivatives) have been shown to be measurable by ELISA and vary depending on disease conditions, although bioanalytical characterization of the assay has been lacking^61^. Previously, proNGF levels have been shown to increase during gestation in murine uterus tissues however as was seen with interspecies variation in NGF levels, it may be difficult to compare proNGF levels in terms of absolute circulating values between species^45^. proNGF levels certainly did not increase in human pregnancy as has been observed in rats^45^, highlighting the potential general difference in the role of proNGF in pregnancy between species. However, this also highlights the need to use carefully qualified assays to affirm that observed changes in mature NGF or proNGF levels are not confounded by bioanalytical issues.

Here we establish a sensitive and selective LC–MS/MS method for the detection of tNGF and proNGF that can be measured simultaneously, which provides specificity to the measurement of these neurotrophins in their various roles as biomarkers in gestation, nervous system development, and disease states compared to currently utilized technologies.

## Materials & methods

### Materials

Lyophilized recombinant human β-Nerve Growth Factor (rhβ-NGF) (Catalog # 256-GF-100; R&D Systems, Minneapolis, MN) was prepared in 0.2% bovine serum albumin (Catalog # Millipore-Sigma, Burlington, MA) in 10 mM phosphate buffered saline, pH 7.4. Lots of human serum from BioIVT were pooled (Various; Westbury, NY). The stable isotope labeled peptides NGF- H2N-AWRFIRIDTACVC**V**^**[+7 Da]**^**L**^**[+6 Da]**^SRKAVRRA-OH (New England Peptides, Gardner, MA), and proNGF—H2N-LRSPRVLFSTQPP**R**^**[+10 Da]**^EAADT-OH (Thermo Fisher Scientific, Waltham, MA) were quantified by amino acid analysis, stored at 5 nmol/mL at −80 °C and used to generate the internal standard peptide stock solution at concentrations of 6.67 and 1.67 pmol/µL, respectively, and further diluted to 0.90 and 0.23 fmol/µL respectively. Goat polyclonal anti-β-NGF antibody (Catalog # AF-256 R&D Systems, Minneapolis, MN) was biotinylated using EZ-link Sulfo-NHS-LC-biotin (Pierce Protein Research Products/Thermo Scientific; Rockford, IL) in PBS. Ligand affinity purified rabbit polyclonal antibody against the tryptic NGF-derived peptide IDTACVCVLSR was obtained from New England Peptide (Gardner, MA), and the proNGF ligand affinity purified rabbit polyclonal antibody against the tryptic proNGF-derived peptide VLFSTQPPR was obtained from ThermoFisher Scientific (Waltham, MA). Antipeptide antibody columns were prepared as noted previously with the antibodies (approximately 1.0 mg per peptide) conjugated to protein-G (Poros-G Applied Biosystems, Bedford, MA) using dimethyl pimelimidate (Pierce Protein Research Products/Thermo Scientific; Rockford, IL) for cross-linking. Surrogate matrix (1% milk solution) was prepared by dissolving 0.5 g powdered milk in 50 mL of sterile Milli-Q Water and further diluted to working solutions in surrogate matrix.

### Analytical methods

Surrogate matrix in sterile Milli-Q Water was prepared. Human serum was used as the QC for NGF, and proNGF QC low. Previously analyzed, pooled lots of human serum from BioIVT were used as the proNGF QC high as well as in the proNGF QC mixing scheme described above.

An aliquot of 200 μL of serum, calibrants, or quality control (QC) sample was added to an Eppendorf LoBind deep well plate, following dilution with 650 μL 0.5% BSA in 10 mM PBS. 10 μL of biotinylated anti-NGF antibody were added to each sample and the plate was sealed and shaken at 4  C overnight (550 rpm). Dynabeads Streptavidin MyOne C1 were resuspended and washed once using 0.05% Tween 20 in PBS. 25 μL of C1 beads were added to each sample and allowed to incubate at room temperature for 45 min with shaking (1000 rpm). Samples were then placed on the Hamilton Star for bead processing. The sample and resuspended beads were transferred to a 0.5 mL Protein Lobind collection plate in 5 × 300 µL transfers (including final wash/transfer). During each transfer the samples were allowed to rest on a magnetic stand for 4 min and the supernatant was aspirated to waste. Two sequential washes with 300 µL 0.1% CHAPS in PBS, and final wash with 300 µL 10 mM PBS, were performed, with the samples allowed to rest on a magnetic stand for 4 min and supernatant aspirated to waste. Proteins were eluted with 140 µL of 30 mM HCl in 5% ACN in water, mixed for 5 min, rest on a magnetic stand for 5 min, and the supernatant was transferred to the final collection plate and neutralized with 35 µL 1 M TRIS, pH 8.3. Ten µL of internal standard working solution was added to each well. Final workup included adding 15 µL of 75 mM TCEP reducing solution (prepared fresh prior to addition) and incubating at 60 °C for approximately 45 min. The plate was allowed to cool to RT for approximately 10 min; and 15 µL of 150 mM IAA were added to each sample well and incubated in the dark at RT for approximately 35 min. Finally, the sample digestion was carried out with 10 µL of 100 µg/mL LysC/Trypsin solution at 37 °C overnight.

### LC–MS/MS methods

85 µL was injected onto a HPLC system containing an anti-peptide antibody column to enrich the tryptic peptides of interest prior to reverse phase nanoflow chromatography comprised of Ultimate 3000 (ThermoFisher Scientific) with the following modules: WPS-3000RS Temperature Controlled autosampler fitted with a 250 µL sample loop; HPG-3400RS Rapid Separation Binary Pump (Micro Pump); NCS-3500RS Nano LC system (Loading pump and Nano Pump); TCC-3200RS (antibody column) column oven; NCS 3500 RSC column oven that encloses valve 2, 3 and the trap column (Thermo µ-Precolumn Cartridge P/N 164649) fitted with an Acclaim PepMap100 C18, 5 µm, 100 Å 300 µm i.d. × 5 mm (60 °C). The LC was connected to a Thermo Quantiva Triple Quadrupole with a Thermo Fisher Easy Spray Ionization Source. The analytical column is Thermo Easy Spray PepMap C18, 75 µm × 15 cm (p/n ES800) (60 °C). MRM transitions monitored in the LC–MS/MS assay for both proteins can be found in Table S5.

### Method qualification

The assay was calibrated with standards at 0, 10, 20, 40, 80, 160, 320, and 640 pg/mL NGF in 1% milk (n = 2). NGF concentrations in QC samples or unknowns were back calculated in TraceFinder 4.1 General Quan using a linear regression analysis (weighted: 1/ × 2) applied to the calibration curve. Precision and accuracy of the tNGF analysis was tested on 3 different days at four levels and with six replicates using QC1 (0.5 × endogenous), QC2 (endogenous tNGF levels, unspiked), QC3 (endogenous + 45 pg/mL NGF), and QC4 (endogenous + 450 pg/mL NGF). A stability assessment was made over 1 freeze–thaw cycle for QC3 and QC4 at − 70 °C (n = 6); and at RT for 4 h at levels of QC3 and QC4 (n = 6).

For the analytical assessment of the pregnancy cohort run; four replicates of each calibration standards were distributed across the two 96-well plates, and the quality control samples at levels of QC2; QC3 and QC4 were split amongst both plates with an n of 8 for each level (n of 4 on each plate).

A relative quantification approach for proNGF was used which did not include the use of a calibration standard. Instead, an ad-mixing scheme was devised to measure lots of human serum that were high (proQCH)and low (proQCL) for proNGF. The Millipore-Sigma serum was used for proQCL and the proQCH was made by combining BioIVT serum lots that were separately determined to have high proNGF levels. The three medium QC levels were made by mixing the proQCL and the proQCH at different ratios: proQCM1: 75% proQCL and 25% proQCH; proQCM2 50% proQCL and 50% proQCH; proQCM3 25% proQCL and 75% proQCH. The proNGF measurement was conducted by establishing the ratio of the light peptide signal area to that of the heavy (stable isotope labelled) peptide.

### Study design and statistical methods

There were four cohorts in this study including pregnant females providing sample across three trimesters (Group I: first trimester, weeks 1–13, n = 24; Group II: second trimester, weeks 14–26, n = 28; Group III: third trimester, weeks 27–40, n = 22) with samples taken near the midpoint of each trimester and the control samples (Group IV: non-pregnant, n = 29). The control non-pregnant cohort was chosen to match as closely as possible to the pregnancy cohorts. The members of the pregnancy cohorts are of Caucasian ethnicity, > 18 years of age, with pregnancy confirmed by HCG test and/or ultrasound. Samples come from a separate cohort of women at each pregnancy stage. Subjects were excluded if they had a history of illicit drug use, current infections including HIV, zika, blood borne sexually transmitted diseases, sickle cell anemia or any other known disease that may impact the study physiologically. Previous infections of Hepatitis B/C, HIV-1, HIV-2, syphilis, and tuberculosis were also excluded. Information about the outcome of pregnancy such as life birth, singleton or twins, was unavailable. The serum was collected by Cureline, Inc. (Brisbane, CA USA) with ethics approval obtained from the WCG Institutional Review Board (WCG IRB Reference No.: 20140236; Cureline Study No. 1144700). This IRB is in full compliance with good clinical practices as defined under the U.S. food and drug administration (FDA) regulations, U.S. department of health and human services (HHS) regulations, and the international conference on harmonization (ICH) guidelines. The participants provided their written informed consent that their samples can be used in biomedical research. All analytical methods were carried out in accordance with relevant Pfizer guidelines and best practices.

tNGF concentrations in serum healthy subjects was previously determined to have a coefficient of variation of about 30%^55^. Therefore, this study was designed and powered to detect a 25–30% difference of tNGF concentration between two groups: requiring a sample size of about 20–25 per group at 80% power. Statistical analyses were done to compare the second and third trimesters with the first, as well as the linear trends both across the semesters as well as all four groups. Log transformation was applied to tNGF to stabilize its variability across groups prior to statistical analysis. Effect of pregnancy was analyzed in a one-way analysis of variance (ANOVA) model. Potential covariates were examined and adjusted if they were significantly associated with tNGF or proNGF. Comparisons between different trimesters and non-pregnant females as well as linear trend across trimesters were tested using contrasts under the ANOVA model. No adjustments for multiple comparisons were required and the statistical significance was achieved when *p* values were less than 0.05 (See Fig. 3). All Analyses were performed in R 3.5.0.

## Supplementary Information

Supplementary Information.

## Data Availability

The datasets generated during and/or analyzed during the current study are available from the corresponding author on reasonable request.
