# Supplementary material for: Analysis of β-nerve growth factor and its precursor during human pregnancy by immunoaffinity-liquid chromatography tandem mass spectrometry
Source: Sci Rep. 2023 Jun 6;13:9180. doi: 10.1038/s41598-023-34695-7 (PMC10244379; doi:10.1038/s41598-023-34695-7)
Supplement: Supplementary file 1 — Supplementary Information. [file 41598_2023_34695_MOESM1_ESM.pdf]

## Supplemental Information

## Supplemental Human Pregnancy Cohort Results

Statistical analysis of the tNGF QCs (Table S1) and proNGF QCs (Table S2) for the pregnancy cohort run can be found below. Individual concentrations for tNGF can be found in Table S3 below. Individual proNGF Light to Heavy Peak Area Ratio can be found in Table S4 below. Figure S1 (Age related Data analysis) and Table S5 (MRM Transitions) can be found below.

**Table S1: NGF Concentrations from Quality Control Samples for the Human Pregnancy Cohort Analysis**

| tNGF | | | |
|---|---|---|---|
| **Level** | **QC2** | **QC3** | **QC4** |
| **Nominal** | **Endogenous** | **Endogenous +45 pg/ml** | **Endogenous +450 pg/ml** |
| **Rep 1** | 28.7* | 58.8 | 479 |
| **Rep 2** | 22.4 | 66.6 | 409 |
| **Rep 3** | 16.9 | 66.7 | 470 |
| **Rep 4** | 25.1 | 86.2* | 451 |
| **Rep 5** | 18.3 | 51.1 | 408 |
| **Rep 6** | 20.3 | 56.8 | 395 |
| **Rep 7** | 22.6 | 52.3 | 392 |
| **Rep 8** | 18.2 | 71.9 | 463 |
| **Avg** | 20.5 | 60.6 | 433 |
| **SD** | 2.96 | 7.96 | 35.7 |
| **CV %** | 14.4 | 13.1 | 8.23 |
| **RE %** | --- | -7.53 | -7.90 |
| ***Inaccuracy >25%**<br>**All points included in statistical calculations** | | | |

**Table S2: proNGF Concentrations from Quality Control Samples for the Human Pregnancy Cohort Analysis**

| proNGF | | | |
|---|---|---|---|
| **Level** | **proQCL** | **QC3** | **QC4** |
| **% Composition**<br>**Units** | **100% proQCL**<br>**[L:H PAR]** | **50% proQCL 50% proQCH**<br>**[L:H PAR]** | **100% proQCH**<br>**[L:H PAR]** |
| **Rep 1** | 0.036 | 0.064 | 0.115 |
| **Rep 2** | 0.033 | 0.069 | 0.094 |
| **Rep 3** | 0.045 | 0.069 | 0.116 |
| **Rep 4** | 0.038 | 0.061 | 0.116 |
| **Rep 5** | 0.029 | 0.061 | 0.115 |
| **Rep 6** | 0.024* | 0.059 | 0.114 |
| **Rep 7** | 0.044 | 0.043* | 0.115 |
| **Rep 8** | 0.037 | 0.064 | 0.096 |
| **Avg** | 0.0374 | 0.0639 | 0.110 |
| **SD** | 0.006 | 0.004 | 0.009 |
| **CV %** | 15.2 | 6.16 | 8.51 |
| **RE %** | --- | -13.4 | |
| ***Accuracy >25%**<br>**All points included in statistical calculations. L:H PAR = Light : Heavy Peak Area Ratio** | | | |

**Table S3: Serum tNGF Concentrations from the Human Pregnancy Cohort**

| Pregnancy Sample Unknowns: NGF | | | | | | | |
|---|---|---|---|---|---|---|---|
| **Trimester 1** | | **Trimester 2** | | **Trimester 3** | | **Control** | |
| **Subject** | **pg/mL** | **Subject** | **pg/mL** | **Subject** | **pg/mL** | **Subject** | **pg/mL** |
| T1_1 | 35.4 | T2_1 | 74.6 | T3_1 | 62.5 | C_1 | 42.5 |
| T1_2 | 59.7 | T2_2 | 55.1 | T3_2 | 63.7 | C_2 | 25.6 |
| T1_3 | 41.5 | T2_3 | 45.4 | T3_3 | 64.6 | C_3 | 41.3 |
| T1_4 | 56.2 | T2_4 | 51.1 | T3_4 | 85.4 | C_4 | 51.9 |
| T1_5 | 32.1 | T2_5 | 57.0 | T3_5 | 76.2 | C_5 | 41.5 |
| T1_6 | 40.3 | T2_6 | 74.4 | T3_6 | 69.3 | C_6 | 57.5 |
| T1_7 | 36.2 | T2_7 | 65.6 | T3_7 | 58.8 | C_7 | 46.5 |
| T1_8 | 42.4 | T2_8 | 57.8 | T3_8 | 63.0 | C_8 | 42.0 |
| T1_9 | 38.7 | T2_9 | 58.1 | T3_9 | 79.0 | C_9 | 35.0 |
| T1_10 | 39.0 | T2_10 | 49.8 | T3_10 | 100.1 | C_10 | 61.8 |
| T1_11 | 34.8 | T2_11 | 68.8 | T3_11 | 96.7 | C_11 | 35.1 |
| T1_12 | 38.5 | T2_12 | 60.7 | T3_12 | 126.7 | C_12 | 82.4 |
| T1_13 | 53.1 | T2_13 | 72.0 | T3_13 | 72.4 | C_13 | 47.9 |
| T1_14 | 64.8 | T2_14 | 47.0 | T3_14 | 75.1 | C_14 | 40.9 |
| T1_15 | 47.0 | T2_15 | 42.1 | T3_15 | 77.2 | C_15 | 55.9 |
| T1_16 | 41.3 | T2_16 | 78.9 | T3_16 | 74.1 | C_16 | 39.5 |
| T1_17 | 34.3 | T2_17 | 51.9 | T3_17 | 59.4 | C_17 | 45.9 |
| T1_18 | 42.1 | T2_18 | 71.7 | T3_18 | 99.5 | C_18 | 66.6 |
| T1_19 | 29.8 | T2_19 | 67.6 | T3_19 | 68.2 | C_19 | 52.2 |
| T1_20 | 31.2 | T2_20 | 64.4 | T3_20 | 77.9 | C_20 | 49.4 |
| T1_21 | 45.5 | T2_21 | 49.5 | T3_21 | 48.9 | C_21 | 36.3 |
| T1_22 | 45.6 | T2_22 | 58.5 | T3_22 | 95.0 | C_22 | 54.3 |
| T1_23 | 37.8 | T2_23 | 60.1 | | | C_23 | 34.4 |
| T1_24 | 56.2 | T2_24 | 71.2 | | | C_24 | 37.5 |
| | | T2_25 | 76.5 | | | C_25 | 31.4 |
| | | T2_26 | 84.2 | | | C_26 | 26.8 |
| | | T2_27 | 85.3 | | | C_27 | 35.7 |
| | | T2_28 | 132.5 | | | C_28 | 37.2 |
| | | | | | | C_29 | 38.3 |

**Table S4: proNGF Concentrations from Unknown Cohort**

| Pregnancy Sample Unknowns: proNGF | | | | | | | |
|---|---|---|---|---|---|---|---|
| Trimester 1 | | Trimester 2 | | Trimester 3 | | Control | |
| Subject | proNGF L:H Ratio | Subject | proNGF L:H Ratio | Subject | proNGF L:H Ratio | Subject | proNGF L:H Ratio |
| T1_1 | 0.157 | T2_1 | 0.114 | T3_1 | 0.214 | C_1 | 0.172 |
| T1_2 | 0.133 | T2_2 | 0.13 | T3_2 | 0.159 | C_2 | 0.205 |
| T1_3 | 0.158 | T2_3 | 0.148 | T3_3 | 0.252 | C_3 | 0.15 |
| T1_4 | 0.169 | T2_4 | 0.164 | T3_4 | 0.178 | C_4 | 0.207 |
| T1_5 | 0.161 | T2_5 | 0.176 | T3_5 | 0.13 | C_5 | 0.206 |
| T1_6 | 0.165 | T2_6 | 0.217 | T3_6 | 0.162 | C_6 | 0.263 |
| T1_7 | 0.153 | T2_7 | 0.171 | T3_7 | 0.152 | C_7 | 0.154 |
| T1_8 | 0.132 | T2_8 | 0.222 | T3_8 | 0.172 | C_8 | 0.12 |
| T1_9 | 0.106 | T2_9 | 0.089 | T3_9 | 0.21 | C_9 | 0.169 |
| T1_10 | 0.171 | T2_10 | 0.15 | T3_10 | 0.21 | C_10 | 0.168 |
| T1_11 | 0.132 | T2_11 | 0.113 | T3_11 | 0.237 | C_11 | 0.155 |
| T1_12 | 0.19 | T2_12 | 0.177 | T3_12 | 0.169 | C_12 | 0.198 |
| T1_13 | 0.101 | T2_13 | 0.146 | T3_13 | 0.185 | C_13 | 0.147 |
| T1_14 | 0.146 | T2_14 | 0.19 | T3_14 | 0.197 | C_14 | 0.18 |
| T1_15 | 0.103 | T2_15 | 0.167 | T3_15 | 0.133 | C_15 | 0.109 |
| T1_16 | 0.117 | T2_16 | 0.173 | T3_16 | 0.154 | C_16 | 0.104 |
| T1_17 | 0.129 | T2_17 | 0.154 | T3_17 | 0.189 | C_17 | 0.181 |
| T1_18 | 0.123 | T2_18 | 0.152 | T3_18 | 0.211 | C_18 | 0.175 |
| T1_19 | 0.137 | T2_19 | 0.148 | T3_19 | 0.127 | C_19 | 0.127 |
| T1_20 | 0.133 | T2_20 | 0.176 | T3_20 | 0.168 | C_20 | 0.131 |
| T1_21 | 0.238 | T2_21 | 0.147 | T3_21 | 0.151 | C_21 | 0.186 |
| T1_22 | 0.173 | T2_22 | 0.142 | T3_22 | 0.153 | C_22 | 0.144 |
| T1_23 | 0.116 | T2_23 | 0.165 | | | C_23 | 0.165 |
| T1_24 | 0.143 | T2_24 | 0.206 | | | C_24 | 0.137 |
| | | T2_25 | 0.232 | | | C_25 | 0.13 |
| | | T2_26 | 0.184 | | | C_26 | 0.182 |
| | | T2_27 | 0.165 | | | C_27 | 0.181 |
| | | T2_28 | 0.167 | | | C_28 | 0.149 |
| | | | | | | C_29 | 0.999 |

## Supplemental Study Design and Statistical Methods

**Figure S1: Age related analysis of NGF and proNGF. No significant association between the age of the individual subjects and levels of either peptide in the various trimesters were observed.**

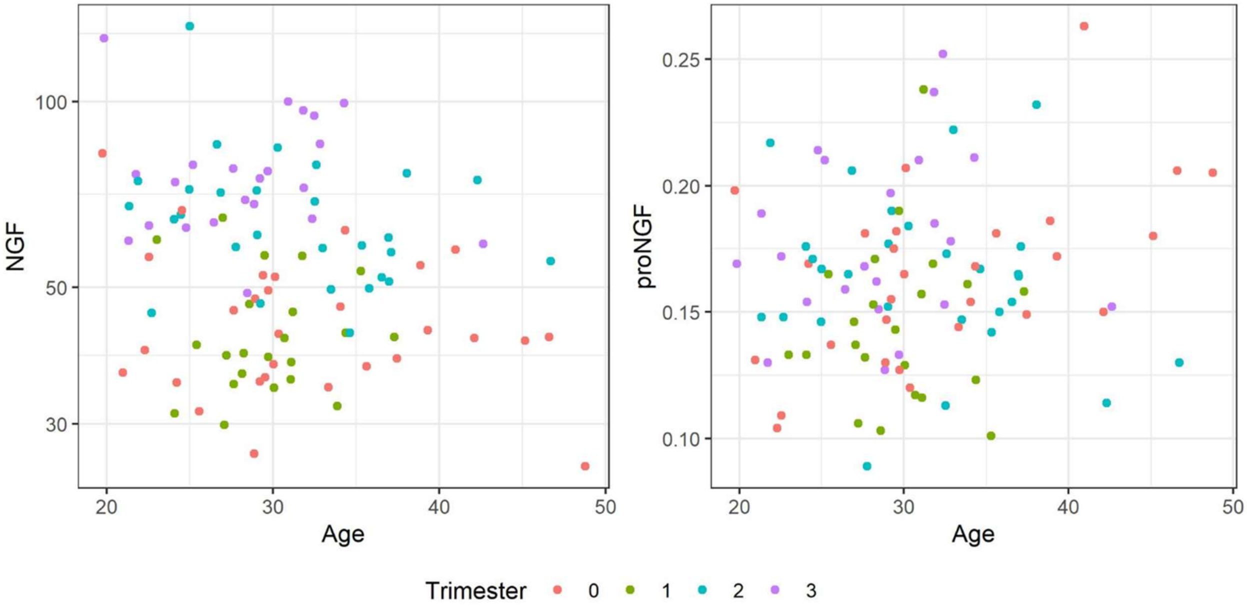

## Supplemental LC-MS/MS Methods

**Table S5: MRM Transition list for NGF, NGF-IS (H) and proNGF, proNGF-IS (H)**

| Compound | Start Time (min) | End Time (min) | Polarity | Precursor (m/z) | Product (m/z) | Fragment | CE (V) |
|---|---|---|---|---|---|---|---|
| proNGF: VLFSTQPPR | 7 | 14 | Positive | 522.795 | 832.431 | y7 | 20.1 |
| proNGF: VLFSTQPPR | 7 | 14 | Positive | 522.795 | 685.363 | y6 | 20.1 |
| proNGF: VLFSTQPPR | 7 | 14 | Positive | 522.795 | 369.224 | y3 | 20.1 |
| proNGF VLFSTQPPR (H) | 7 | 14 | Positive | 527.799 | 842.439 | y7 | 20.1 |
| proNGF VLFSTQPPR (H) | 7 | 14 | Positive | 527.799 | 695.371 | y6 | 20.1 |
| proNGF VLFSTQPPR (H) | 7 | 14 | Positive | 527.799 | 379.233 | y3 | 20.1 |
| NGF: IDTACVCVLSR | 7 | 14 | Positive | 647.318 | 964.47 | y8 | 24.3 |
| NGF: IDTACVCVLSR | 7 | 14 | Positive | 647.318 | 893.433 | y7 | 24.3 |
| NGF: IDTACVCVLSR | 7 | 14 | Positive | 647.318 | 733.403 | y6 | 24.3 |
| NGF: IDTACVCVLSR (H) | 7 | 14 | Positive | 653.833 | 977.501 | y8 | 24.3 |
| NGF: IDTACVCVLSR (H) | 7 | 14 | Positive | 653.833 | 906.464 | y7 | 24.3 |
| NGF: IDTACVCVLSR (H) | 7 | 14 | Positive | 653.833 | 746.433 | y6 | 24.3 |

**Min: Minute; m/z: mass to charge ratio; CE: collision energy; V: voltage**
